# Evaluation of serum C-reactive protein and high mobility group box 1 concentrations in 22 dogs with acute pancreatitis: a pilot study

**DOI:** 10.1080/01652176.2019.1655178

**Published:** 2019-08-10

**Authors:** Hakhyun Kim, Hyung-Jin Kim, Ji-Houn Kang, Byeong-Teck Kang, Mhan-Pyo Yang

**Affiliations:** Veterinary Medical Center and College of Veterinary Medicine, Chungbuk National University, Cheongju, Chungbuk, Republic of Korea

**Keywords:** Dog, canine, pancreatitis, HMGB1, SIRS

## Abstract

**Background:** High mobility group box 1 (HMGB1) is an important mediator of systemic inflammatory response syndrome (SIRS) in humans with severe acute pancreatitis (AP), but there is little information regarding its role in dogs.

**Aim:** To compare the serum concentrations of C-reactive protein (CRP) and HMGB1 in healthy dogs and those with AP with or without SIRS.

**Methods:** The study included 22 dogs with AP and 20 healthy dogs. CRP and HMGB1 were assessed by ELISA. Statistical analyses were conducted by non-parametric tests.

**Results:** Median (interquartile range) serum CRP and HMGB1 concentrations were significantly (*P* < 0.05) higher in dogs with AP [60.56 (14.50–140.10) µg/mL and 0.35 (0.03–1.12) ng/mL, respectively] than in healthy dogs [2.23 (1.75–5.14) µg/mL and 0.02 (0.01–0.05) ng/mL, respectively]. After the recommended treatments for AP, serum CRP concentration in AP dogs significantly decreased, but that of HMGB1 in AP dogs significantly increased. There was also a significant difference in median serum HMGB1 concentration between AP dogs with and without SIRS. The use of serum HMGB1 concentration of 0.35 ng/mL to distinguish AP dogs with and without SIRS was associated with a sensitivity of 87.5% and a specificity of 71.5%. A positive correlation was identified between HMGB1 and clinical severity of AP. All AP dogs had a positive outcome during hospitalization [6.0 (1.5–6.0) days].

**Conclusion:** Results indicate that HMGB1 might be a useful biomarker for the progression of AP and may play a role in progression of AP into SIRS in dogs.

## 1. Introduction

Acute pancreatitis (AP) develops when there is abnormally early activation of trypsin and other pancreatic proteases within the pancreas that overwhelms both the local safeguards within pancreatic acinar cells and the capacity of antiproteases in the circulatory system (Mansfield 2012a, 2012b). It is caused by oxidative stress, or hypotension and/or impaired local microcirculation (Mansfield 2012a, 2012b). Although many dogs have subclinical or mild disease, AP can progress to become associated with systemic complications, such as systemic inflammatory response syndrome (SIRS) or multiple organ failure, which increases the mortality rate to 27–58% in dogs (Cook et al. 1993; Pápa et al. 2011; Mansfield 2012a). The potential for the development of severe complications and the associated high mortality implies that clinicians should attempt to determine whether a case is mild or severe, using an appropriate method, and institute hospitalization and intensive care immediately if it is considered to be severe (Sato et al. 2017). Various studies have identified biomarkers of AP such as serum paraoxonase 1 activity and C-reactive protein (CRP) (Tvarijonaviciute et al. 2015; Sato et al. 2017). However, to date no reliable biomarkers of severe AP or of the potential for progression from mild to severe AP with systemic complications have been identified.

High mobility group box 1 (HMGB1) is a non-histone chromosomal protein that is passively released from damaged cells or actively released from activated cells, such as inflammatory cells (Jiang et al. 2007). Actively secreted HMGB1 is considered to be a late-phase inflammatory cytokine because it is detected 8–18 h after the initial insult (Uhrikova et al. 2015). Secreted HMGB1 also induces inflammation by stimulating proinflammatory cytokine production and activating inflammatory pathways (Scaffidi et al. 2002; O’Connor et al. 2003). Conversely, inhibition of HMGB1 activity can protect against severe AP and ameliorate the pathology in extra-pancreatic organs, such as lung, liver, intestine, and kidney in experimental animal models of AP (Ulloa et al. 2002; Sawa et al. 2006), suggesting that HMGB1 is an important mediator of the progression from local pancreatic injury to SIRS. Therefore, serum HMGB1 may represent a biomarker of the progression to SIRS in individuals with AP, but this possibility has not been investigated in veterinary medicine (O’Connor et al. 2003) or specifically in dogs with AP yet.

Therefore, we hypothesized that the serum HMGB1 concentration might be higher in AP dogs with SIRS than in those without SIRS. The aim of this study was to compare the serum HMGB1 and CRP concentrations in healthy dogs and dogs with AP as well as in dogs with AP, with or without SIRS.

## 2. Materials and methods

### 2.1. Animals

This was a prospective case-control study. Sixty-five dogs with AP that were newly diagnosed between September 2016 and July 2018 were initially considered. Of these, 23 dogs with recurrent bouts of AP and five dogs referred from other hospitals (that had been diagnosed with AP and hospitalized for >2 days) were excluded. Fifteen dogs with concurrent diseases, including chronic kidney disease (five dogs), protein-losing enteropathy (three dogs), hyperadrenocorticism (two dogs), pneumonia (two dogs), bacterial cystitis (one dog), periodontal disease (one dog), or prostatitis (one dog) were also excluded on the basis of their clinical data. Consequently, 22 dogs with AP were included in the present study. Twenty healthy, client-owned dogs were also included as controls. These animals were recruited from the same veterinary medical center when they presented for examination, and were considered to be clinically healthy on the basis of physical examination, fecal examination, heartworm antigen testing, complete blood count, serum biochemical analysis, serum electrolyte analysis, urinalysis, adrenocorticotropic hormone response testing, and diagnostic imaging (survey radiography and abdominal ultrasonography).

Informed consent was obtained from the owners, and the University Ethics committee (protocol CBNUA-1179-18-01) approved the study.

### 2.2. Diagnosis of AP

A diagnosis of pancreatitis was established only if all the abnormal findings were compatible with acute onset of the disease. These findings comprised abnormalities consistent with pancreatitis on abdominal ultrasound and a positive SNAP canine pancreas-specific lipase (cPL) test (IDEXX Reference Laboratory, Seongnam-si, Korea) (*n* = 13) or highly specific cPL (IDEXX Reference Laboratory, Seongnam-si, Korea) concentration (*n* = 9), as described elsewhere (Trivedi et al. 2011; Mansfield 2012a; Paek et al. 2014). Dogs exhibiting only some of these diagnostic criteria were not included in the study. Clinical evidence of an acute presentation of pancreatitis was defined as acute (<2 days) on the basis of one or more of the following: vomiting, anorexia, and/or abdominal pain during a physical examination on admission, or an established history (Mansfield 2012a). Ultrasonographic findings that were regarded as suggestive of pancreatitis included hypo/hyperechoic lesions, or mixed patterns, in pancreata that appeared to be enlarged and irregularly shaped. In addition, ultrasonographic changes typically recognized as being secondary to pancreatitis, such as hyperechoic mesentery, localized free abdominal fluid, thickening of the duodenal or gastric walls, duodenal spasm, appearance of inflammation in the adjacent intestines, and a dilated common bile duct were also considered to be evidence of pancreatitis (Mansfield 2012a). The results of SNAP cPL tests were interpreted as abnormal only if the color of the sample spot was more intense than that of the reference spot. In the highly specific cPL assays, concentrations >400 μg/L were considered to be consistent with pancreatitis (Xenoulis and Steiner 2012).

### 2.3. Clinical severity of AP

At initial evaluation, all dogs were given a clinical severity score. The clinical severity of AP was assessed considering endocrine, hepatic, renal, hematopoietic, local complications, cardiac, respiratory, intestinal integrity, vascular forces by previously validated method (Mansfield et al. 2008), which assigned a clinical score based on 9 variables (Table 1). After summation of each variable score, the total composite score was determined (potential maximum of 24 points in total).

**Table 1.** Findings associated with various body systems that were assessed as part of a clinical severity for acute pancreatitis in dogs (from Mansfield et al. 2008).

System	Finding	Points
Endocrine	No abnormalities Preexisting diabetes mellitus Diabetic ketoacidosis	0 1 2
Hepatic	No abnormalities ≥2.5-fold increase (compared with upper limit of reference range) in at least 2 of the following: serum alkaline phosphatase, alanine transferase, and aspartate aminotransferase activities ≥5-fold increase (compared with upper limit of reference range) in at least 2 of the following: serum alkaline phosphatase, alanine transferase, and aspartate aminotransferase activities Extrahepatic bile duct obstruction	0 1 2 3
Renal	No abnormalities Azotemia (≤1.5-fold increase [compared with upper limit of reference range] in serum urea and creatinine concentration) Anuria or azotemia (≥1.5-fold increase [compared with upper limit of reference range] in serum urea and creatinine concentration)	0 1 2
Hematopoietic	No abnormalities WBC count ≥ 20.0 × 10^9^ cells/L or ≤ 4.0 × 109 cells/L, with ≤ 10% band neutrophils WBC count ≥ 20.0 × 10^9^ cells/L or ≤ 4.0 × 10^9^ cells/L, neutrophil count ≤ 1.0 × 10^9^ cells/L, or ≥ 10% band neutrophils Clinicopathologic evidence of hypercoagulability or coagulation abnormalities Clinical evidence of disseminated intravascular coagulation or bleeding diathesis	0 1 2 3 4
Local complications	No abnormalities Peritonitis extending beyond peripancreatic area Pseudocyst or other acute fluid accumulation Pancreatic abscess	0 1 2 3
Cardiac	No abnormalities <60 ventricular premature complexes/24-hour period or heart rate >180 beats/min Paroxysmal or sustained ventricular tachycardia	0 1 2
Respiratory	No abnormalities Clinical evidence of dyspnea or tachypnea (>40 breaths/min) Clinical evidence of pneumonia or acute respiratory distress syndrome	0 1 2
Intestinal integrity	No abnormalities Intestinal sounds not detected during > 3 auscultations in 24-h period Hematochezia, melena, or regurgitation No enteral food intake for > 3 days No enteral food intake for > 3 days and at least 2 of the following: hematochezia, melena, and regurgitation	0 1 2 3 4
Vascular forces	No abnormalities Systolic arterial blood pressure < 60 or > 180 mm Hg or serum albumin concentration < 18 g/L Systolic arterial blood pressure < 60 or > 180 mm Hg and serum albumin concentration < 18 g/L	0 1 2

After summation of each system score, the total composite score was determined (potential maximum 24 points in total). WBC, white blood cell.

### 2.4. Treatment

Treatment was administered as recommended in the literature at the time of the study (Steiner 2010; Mansfield 2012a; Mansfield and Beths 2015). The most important component of the treatment was intravenous fluid therapy, but the other prescribed medications comprised analgesics, anti-emetics, broad-spectrum antibiotics, histamine (H_2_) receptor blockers, fresh plasma transfusion, and/or low-molecular weight heparin. *Nil per os* (NPO) was maintained until vomiting stopped. Briefly, intravenous fluid therapy with crystalloids was initiated promptly upon hospitalization to correct dehydration. On the basis of the assumption that abdominal pain is likely to be present in all dogs with AP, butorphanol tartrate (Butophan, Muungmoon Pharm. Co., Seoul, Korea) was administered (0.2 mg/kg BW IV q 6 h). Maropitant citrate (Cerenia, Pfizer, Pocé-sur-Cisse, France), which blocks centrally and peripherally mediated emesis (1 mg/kg BW SC q 24 h) was used as an anti-emetic. Bacterial complications are rare in dogs with AP, but the dogs were treated with broad-spectrum antibiotics if pyrexia, left-shifted neutropenia, or documented infection was present (Mansfield 2012a). Famotidine (Gaster Inj., Dong-A ST, Seoul, Korea), an H_2_ receptor blocker, was also administered (0.5 mg/kg BW IV q 12 h) if melena, hematochezia or hematemesis was present. Dogs were maintained NPO if vomiting continued despite the anti-emetic therapy, whereas if the vomiting stopped, water was reintroduced slowly, followed by small amounts of a low-fat diet (Royal Canin Veterinary Diet Gastrointestinal Low Fat Canned Dog Food; Royal Canin, St. Charles, USA) the following day.

### 2.5. Grouping

Dogs with AP were categorized as ‘SIRS’ or ‘non-SIRS’ depending on whether they satisfied at least two out of four SIRS criteria at presentation. The SIRS criteria used in the present study were hypothermia [rectal body temperature < 100.6 °F] or hyperthermia [rectal body temperature > 102.6 °F]; heart rate > 140 beats/min; respiratory rate > 20 breaths/min; and leukopenia (white blood cell count < 6.3 × 10^9^/L), leukocytosis (white blood cell count > 16.3 × 10^9^/L), or >3% band neutrophils (Hauptman et al. 1997).

Dogs with AP were discharged following obvious improvement or recovery during hospitalization. Evidence of the effective treatment of dogs with AP included the resolution of clinical signs consistent with AP and a negative result on the SNAP cPL test (Kim et al. 2017).

### 2.6. Assays

Blood samples were collected from dogs with AP (*n* = 22) and controls upon admission (*n* = 20), and also from dogs with AP following treatment (*n* = 9), at the time their clinical signs resolved. Blood was collected from the jugular or a peripheral vein, serum was separated from clotted whole blood by centrifugation at 1200 × *g* for 10 min, within 1 h of blood collection, and the serum was stored at −80 °C until assayed.

Serum CRP concentration was measured using a canine-specific ELISA kit (Canine C-Reactive Protein ELISA Kit, BD Biosciences, San Jose, USA), according to the manufacturer’s protocol; the intra-assay variability was <5%, the inter-assay variability was <10%, and the detection limit was 0.015 µg/mL. Because the amino acid sequence of HMGB1 is highly conserved among species, with 100% homology between humans and dogs (Murua Escobar et al. 2003), serum HMGB1 concentration was measured using a human HMGB1 ELISA kit (Human HMGB1 ELISA Kit, MyBiosource Inc., San Diego, USA), for which the intra- and inter-assay variabilities were <4.2% and <8.2%, respectively. The detection limit was 0.01 ng/mL. All samples, standards and controls were assayed in duplicate. The optical density was determined at 450 nm using an automated microplate reader (ELx 808, BioTek Instruments Inc., Winooski, USA).

### 2.7. Statistical analyses

Data were analyzed using commercially available statistical software (Prism 6.01, GraphPad Software Inc., La Jolla, USA). Data are expressed as median (interquartile range). *P* values were calculated for two-tailed tests, and the 95% confidence intervals (CIs) for the differences between medians were determined. The D'Agostino–Pearson omnibus test was performed to determine whether data were normally distributed, and following statistical analyses were conducted by using non-parametric tests because many groups did not follow a normal distribution. The Mann–Whitney *U* test was used to compare the differences between the AP and control dogs or AP dogs with and without SIRS. The Wilcoxon-signed rank sum test was used to compare data for the AP dogs before and after AP treatment. The receiver operator characteristics area under the curve was used to assess the optimal cut-off value of HMGB1 concentration for the differentiation of dogs with AP that did or did not progress to SIRS, along with the corresponding sensitivity and specificity values. Commercially available statistical software (Prism 6.01, GraphPad Software Inc., La Jolla, USA) was also used to undertake the Receiver Operating Characteristic analysis. The relationships between variables were evaluated using Spearman’s correlation. *P* < 0.05 was considered to represent statistical significance.

## 3. Results

### 3.1. Study population

Twenty-two AP dogs [five intact females, seven spayed females, four intact males, and six castrated males; median (range) age, 13.0 (4.0–18.0) years; median (range) weight, 3.8 (1.36–3.50) kg; body condition score, 2–6/9] and 20 healthy dogs [seven intact females, four spayed females, six intact males, and three castrated males; median (range) age, 11.5 (5.5–14.0) years; median (range) weight, 2.94 (1.82–7.94) kg; body condition score, 3–6/9] were included in this study. Among the dogs with AP, 5 were Malteses, 3 were Shih Tzus, 3 were Yorkshire Terriers, 2 were Cocker Spaniels, 2 were Miniature Poodles, 2 were Miniature Schnauzers, 2 were Pomeranians, and 1 each was a Beagle, Pekingese, and Cross breed. Among the healthy dogs, 6 were Beagles, 5 were Malteses, 3 were Shih Tzus, 3 were Miniature Poodles, 2 were Yorkshire Terriers, and 1 was a Miniature Schnauzer. AP and healthy dogs did not significantly differ in terms of age, body weight, or body condition score. All AP dogs had a positive outcome during hospitalization [median (interquartile range) time of hospitalization, 6.0 (1.5–6.0) days].

### 3.2. Serum concentrations of CRP and HMGB1 in dogs with AP and healthy dogs at admission

**Figure 1.**
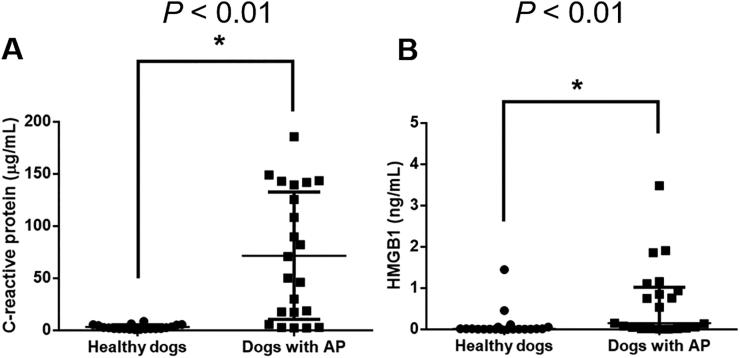
Comparison of the serum concentrations of (A) C-reactive protein and (B) HMGB1 between dogs with AP (*n* = 22) and healthy dogs (*n* = 20) at hospital admission. The horizontal bars indicate the medians and interquartile ranges. *P < 0.05 (Mann–Whitney *U* test). HMGB1, high mobility group box 1.

The median (interquartile range) of the serum CRP concentration was significantly higher (*P* < 0.01) in dogs with AP [60.56 (14.50–140.10) µg/mL] than in healthy dogs [2.23 (1.75–5.14) µg/mL] (Figure 1). The median (interquartile range) of the serum HMGB1 concentration was also significantly higher (*P* < 0.01) in dogs with AP [0.35 (0.03–1.12) ng/mL] than in healthy dogs [0.02 (0.01–0.05) ng/mL].

### 3.3. Difference in the serum concentrations of CRP and HMGB1 in AP dogs with and without SIRS at admission

**Figure 2.**
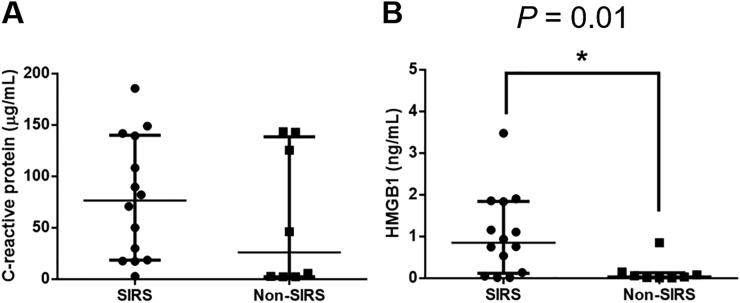
Comparison of the serum concentrations of (A) C-reactive protein and (B) HMGB1 between SIRS (*n* = 14) and non-SIRS (*n* = 8) dogs with AP at admission. The horizontal bars indicate the medians and interquartile ranges. *P < 0.05 (Mann–Whitney *U* test). HMGB1, high mobility group box 1; SIRS, systemic inflammatory response syndrome.

AP dogs were categorized into ‘SIRS’ (*n* = 14) or ‘non-SIRS’ (*n* = 8) subgroups. Only dogs fulfilling SIRS criteria at admission were included in the SIRS subgroup. There was no significant difference in the serum CRP (*P* = 0.29) concentration between the SIRS and non-SIRS subgroups (Figure 2). However, the median (interquartile range) of the serum HMGB1 concentration was significantly higher (*P* = 0.01) in the SIRS subgroup [0.85 (0.12–1.85) ng/mL] than in the non-SIRS subgroup [0.05 (0.01–0.13) ng/mL].

### 3.4. Receiver operating characteristic curve of the serum HMGB1 concentration in AP dogs with and without SIRS

**Figure 3.**
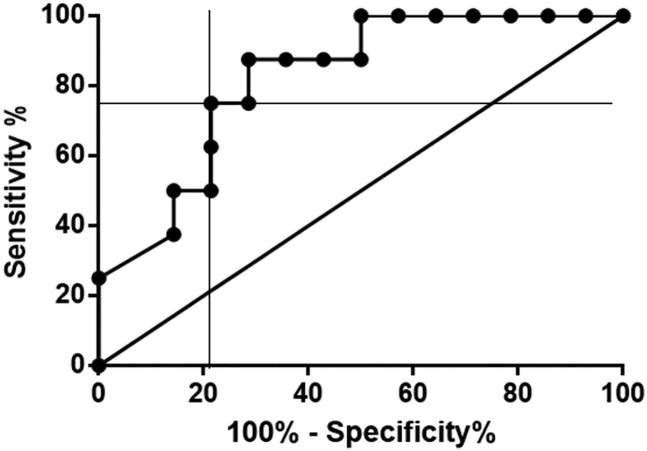
Receiver operating characteristic curve illustrating the sensitivity and specificity for the use of HMGB1 to distinguish dogs with AP and SIRS from those with AP but no SIRS. The thick diagonal line represents a completely uninformative test, wherein the area under curve is 50%. The area under the curve of the receiver operating characteristic curve represented by the thicker line is 0.82 (95% CI = 0.6462–0.9967). The point of intersection indicates the optimal cut-off of 0.35 ng/mL for the differentiation between AP dogs with and without SIRS, with corresponding sensitivity and specificity of 87.5% (47.35–99.68%) and 71.43% (41.90–91.61%). AP, acute pancreatitis; HMGB1, high mobility group box 1; SIRS, systemic inflammatory response syndrome.

The ROC area under the curve was 0.82 (95% CI = 0.6462–0.9967) (Figure 3). The optimal serum HMGB1 cut-off between SIRS and non-SIRS dogs with AP was determined graphically to be 0.35 ng/mL, with the area under the curve being 0.82, the sensitivity 87.5% (95% CI = 47.35–99.68%), and the specificity 71.4% (95% CI = 41.90–91.61%). At a cut-off point of < 0.07 ng/mL, sensitivity and specificity were 62.5% (95% CI = 24.49–91.48%) and 78.57% (95% CI = 49.20–95.34%). At a cut-off point of < 0.90 ng/mL, sensitivity and specificity were 100.0% (63.06–100.0%) and 50.0% (23.04–76.96%).

### 3.5. Relationships between CRP and HMGB1, and highly specific cPL and clinical severity in dogs with AP at admission

**Figure 4.**
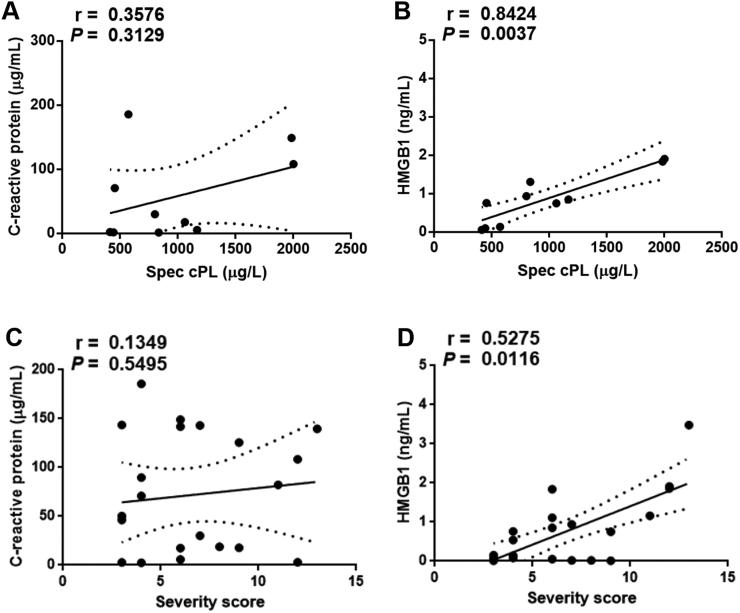
Correlations between the serum concentrations of CRP (A, C) and HMGB1 (B, D), with highly specific cPL (*n* = 9) and the severity score (*n* = 22) in AP dogs. The dotted lines indicate the 95% confidence intervals. *P < 0.05 (Spearman correlation). AP, acute pancreatitis; CRP, C-reactive protein; HMGB1, high mobility group box 1.

It was assessed whether both CRP and HMGB1 were correlated with highly specific cPL (*n* = 9). The serum concentration of HMGB1 was positively correlated with the concentration of highly specific cPL (*r* = 0.84, *P* < 0.01) in dogs with AP (Figure 4). However, no significant correlation was identified between serum CRP concentration and highly specific cPL concentration (*r* = 0.36, *P* = 0.31) in dogs with AP.

All AP dogs (*n* = 22) were assessed according to the clinical severity index of canine AP at admission. The serum HMGB1 concentration was positively correlated with clinical severity of AP (*r* = 0.53, *P* = 0.01) in dogs with AP. However, no significant correlation was identified between the serum CRP concentration and clinical severity of AP (*r* = 0.13, *P* = 0.55) in dogs with AP. In addition, there was no significant correlation between the serum CRP and HMGB1 concentrations (*r* = 0.02, *P* = 0.97) in dogs with AP.

### 3.6. Comparison of serum CRP and HMGB1 concentrations in SIRS dogs with AP before and after treatment

**Figure 5.**
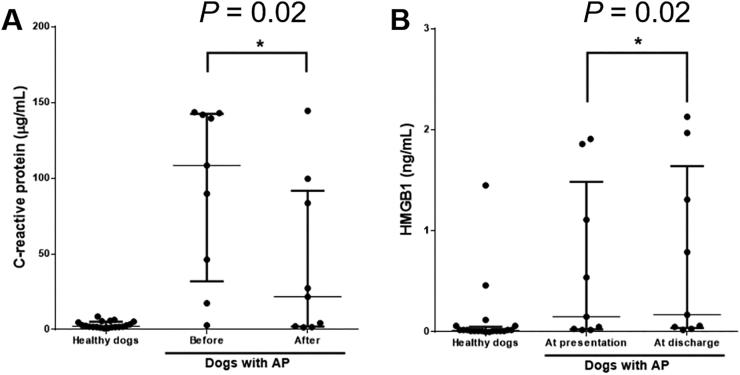
Comparison of serum concentrations of (A) C-reactive protein and (B) HMGB1 before and after treatment in AP dogs with SIRS (*n* = 9). The horizontal bars indicate the medians and interquartile ranges. *P < 0.05 (Wilcoxon matched-pairs signed rank test). AP, acute pancreatitis; HMGB1, high mobility group box 1; SIRS, systemic inflammatory response syndrome.

Five of the fourteen SIRS dogs with AP were not evaluated after treatment because the owners refused permission. In the remaining nine SIRS dogs with AP, the median (interquartile range) of the serum CRP concentration was significantly lower (*P* = 0.02) after treatment [21.73 (1.94–91.62) µg/mL] than before [108.4 (31.86–142.5) µg/mL] (Figure 5). The median (interquartile range) of the serum HMGB1 concentration was significantly higher (*P* = 0.02) after treatment [0.17 (0.04–1.64) ng/mL] than before [0.15 (0.03–1.49) ng/mL].

## 4. Discussion

The present study showed that serum HMGB1 concentration significantly differs between dogs with AP and healthy dogs, as well as between AP dogs with and without SIRS on admission. Moreover, there was a positive correlation between the serum concentrations of HMGB1 and clinical severity of AP in dogs with AP, implying that the former may represent a potential biomarker of the severity of AP (Sato et al. 2017). These findings also suggest that HMGB1 might have a role in the development of AP or the progression of AP to SIRS in dogs. This is the first study to determine the serum concentrations of HMGB1 in dogs with AP.

The finding that serum HMGB1 concentration is higher in dogs with AP than in healthy dogs is consistent with that of a study of humans that demonstrated serum HMGB1 concentration to be significantly higher in severe AP than in healthy volunteers (Yasuda et al. 2006). HMGB1 is highly expressed in the liver, thymus, lymph tissue, testis, and pancreas, but is a ubiquitous nuclear protein in mammals (Erlandsson Harris and Andersson 2004). In a study of experimental AP, the intracellular expression of HMGB1 was initially high in the pancreas, but it was also released into the bloodstream, leading to an increase in serum HMGB1 concentration (Yu et al. 2016). The high serum HMGB1 concentration in dogs with AP is also likely to be the result of its release from pancreatic tissue, although measurement of the mRNA and protein expression of HMGB1 in the canine pancreas will be necessary to confirm this. However, HMGB1 can be released by activated macrophages/monocytes as well as damaged pancreatic cells (Yasuda et al. 2006; Yu et al. 2016). Inflammatory cells appear within pancreatic tissue during inflammation and produce a wide range of cytokines and chemokines, which is referred to as a cytokine storm (Mansfield 2012b). A similar finding has also been made in dogs with AP (Paek et al. 2014). Therefore, macrophages/monocytes may release HMGB1 in dogs with AP in response to cytokine stimulation.

HMGB1 may be involved in the inflammation and necrosis in severe AP and be an important mediator in the pathogenesis of SIRS in humans with AP (Shen and Li 2015). However, there have been no reports regarding HMGB1 concentration in dogs with AP and SIRS. Our results show that the serum HMGB1 concentration of dogs with AP and SIRS is significantly higher than that of dogs with AP but not SIRS. SIRS is a sequela of a number of known diseases, including pancreatitis, and is associated with the widespread release of proinflammatory cytokines in response to infectious or non-infectious insults (Rangel-Frausto et al. 1995). Although its pathogenesis has not been completely described, pancreatic inflammation can be further exacerbated, leading to the development of SIRS, which is characterized by an overwhelming inflammatory response (Shen and Li 2015). In the present study, serum HMGB1 concentration significantly correlated with highly specific cPL activity, which is an accepted short-term prognostic indicator in dogs with pancreatitis (Sato et al. 2017). Although the relationship between highly specific cPL and the severity of AP has not been investigated in dogs, it is possible that a large amount of highly specific cPL leaks from the damaged pancreas in dogs that progress to SIRS (Sato et al. 2017). HMGB1 concentration was significantly higher in SIRS dogs than in non-SIRS dogs, but CRP concentration has previously been shown not to differ between these groups (Ishida et al. 2011), and our findings were consistent with this, which implies that serum HMGB1 may represent a marker of SIRS, and that HMGB1 monitoring might be clinically useful during the treatment of dogs with AP. Early recognition of the development of SIRS would mean that more intensive therapy could be instituted for dogs with AP that are at an early stage of SIRS, which might be associated with a better prognosis. To this end, it would be necessary to measure serum HMGB1 serially in dogs at presentation and during hospitalization.

Dogs are generally discharged on the basis of an obvious improvement in their clinical signs and the return of spontaneous feeding. In the present study, after treatment of the AP, serum CRP concentration had decreased, but serum HMGB1 concentration had significantly increased, from presentation. This may be explained by the differing characteristics of these markers. HMGB1 concentration gradually increases and peaks 72 h after a stimulus, whereas CRP increases more rapidly, peaking at 24 h (Ishida et al. 2011). Therefore, it is possible that the serum HMGB1 concentration was high because of passive release from injured pancreatic tissue at presentation, whereas its active release by neutrophils or other immune cells in response to proinflammatory cytokines resulted in a further increase in circulating levels by the time of discharge. Thus, CRP, an acute-phase protein, might be a more useful biomarker for the occurrence of pancreatic inflammation, but not for the development of SIRS, based on our results. HMGB1, a late-phase protein in inflammation, might be not an indicator of the development of pancreatic inflammation itself, but a more potent indicator of severe complications such as SIRS in dogs. Alternatively, this finding may be explained by the fact that the day of hospital admission (day of sample collection for the study) and the day of the AP condition were not the same in each dog. This is an inherent problem of a clinical study. These results should be interpreted with caution due to the small number (*n* = 9) of AP dogs included in statistical analyses. Study of the kinetics of HMGB1 in a large number of AP dogs, involving serial measurement of the HMGB1 concentration from presentation to hospital discharge, is necessary to evaluate the usefulness of HMGB1 in a clinical setting.

This study had several limitations. The first was the small number of dogs studied, which increases the likelihood of negative findings. Another limitation of the study was that we did not compare the data between survivors and dogs that died. In a study conducted in humans, HMGB1 was higher in patients that died than in survivors (Yasuda et al. 2006). Future studies should perform this comparison to clarify the relationship between serum HMGB1 concentration and the severity of AP in affected dogs. In addition, we did not take into account that all the investigations of HMGB1 could have been influenced by other comorbidities such as obesity (body condition score >4/5 or >6/9), any other conditions (e.g. hypothyroidism and idiopathic hyperlipidemia), and administration of medications (e.g. corticosteroids and antiepileptic drugs) that can potentially trigger AP and affect the serum HMGB1 concentration. It would also have been useful to measure the serum HMGB1 concentration in dogs with comorbidities other than pancreatitis and to differentiate the general effects of AP/SIRS on the serum HMGB1 concentration, compared with the more specific effects of AP/SIRS. Finally, the kinetics of HMGB1 in dogs have not been studied in detail. Further investigation of HMGB1 kinetics may enhance understanding of the role of HMGB1 in dogs with AP.

## 5. Conclusions

In conclusion, the findings of this study demonstrate that the serum concentration of HMGB1 is increased in dogs with AP. This may be the result of passive release from damaged pancreatic cells in the early stage of the disease, supplemented by subsequent active secretion by proinflammatory cells. Furthermore, HMGB1 might play a role in the progression of pancreatic inflammation to SIRS, and is a potential marker to identify the development of SIRS in dogs with AP.
